# Clinical evidence of immunogenicity of CAR-T cell therapies and its implication in the clinical development of CAR-T drug products

**DOI:** 10.3389/fimmu.2025.1512494

**Published:** 2025-02-21

**Authors:** Hammodah R. Alfar, Cecil Chen, Eric Lachacz, Weifeng Tang, Yuqian Zhang

**Affiliations:** ^1^ Department of Molecular and Cellular Biochemistry, College of Medicine, University of Kentucky, Lexington, KY, United States; ^2^ Clinical Pharmacology & Quantitative Pharmacology, Biopharmaceuticals R&D, AstraZeneca, Gaithersburg, MD, United States; ^3^ Clinical Pharmacology & Quantitative Pharmacology, Biopharmaceuticals R&D, AstraZeneca, San Francisco, CA, United States; ^4^ Integrated Bioanalysis, BioPharmaceuticals R&D, AstraZeneca, Mölndal, Sweden

**Keywords:** CAR-T, immunogenicity, anti-drug antibody (ADA), biologics, cellular kinetics, clinical monitoring

## Abstract

Chimeric antigen receptor-engineered T cell therapies (CAR-T) are becoming powerful immunotherapeutic tools for treating malignancies, especially hematological malignancies. Like other biological drugs, CAR-T cell products can trigger unwanted immune responses in patients receiving the treatment. This might lead to treatment failure or life-threatening consequences. This immunogenicity could also affect the CAR-T cells’ cellular kinetics and clinical responses. In this review, we summarize the immunogenicity of biologics and their effects on PK/PD profiles, safety, and efficacy. We also introduce the mechanisms of immunogenicity induced by CAR-T cells and clinical evidence of immunogenicity of the currently FDA-approved CAR-T cell products. Particularly, we summarize the currently available immunogenicity data from each CAR-T cell product’s clinical trials, immunogenicity assays, sample types, and preclinical efficacy models, which were retrieved from the FDA and EMA websites. We also discuss a preclinical model that is promising for evaluating CAR-T cell immunogenicity.

## CAR-T cell therapy

Chimeric antigen receptors (CAR) are fusion proteins that can be expressed on the surface of T cells (so-called CAR-T cells) to redirect their specificity to antigen targets presented on tumor cells (1, 2). These modifications allow CAR-T cells to specifically attack tumor cells without the need for the typical T cell receptor (TCR) - major histocompatibility complex (MHC) interaction (3, 4). Upon interaction with their antigen targets on tumor cells, CAR-T cells are activated through the intracellular signaling domains (3, 4). Once activated, CAR-T cells proliferate, secrete cytokines, infiltrate tumor areas, and release cytolytic granules to eliminate targeted cells through an antigen-dependent process (3, 4). The CAR structure has evolved rapidly to enhance CAR-T cell activity, expansion, persistence, and on-target specificity (3, 4). Most of these improvements have occurred in the intracellular domain (endodomain), which is important for CAR-T cell activation, expansion, cytotoxic response, and cytokine production (3, 4). So far there are five generations of CAR-T cell therapies.

To generate autologous CAR-T cells, the patient’s peripheral blood T cells are isolated and modified *ex-vivo* using gene transfer through viral vectors that are replication-deficient but can integrate the CAR cassette into the T cell’s DNA (3, 4). Transduced cells are then expanded in culture, purified, and infused back into the patient (3, 4). Two major toxicities can arise from CAR-T cell infusion— cytokine release syndrome (CRS) and immune effector cell-associated neurotoxicity syndrome (ICANS; also known as neurotoxicity) as a result of immune cells, platelets, and endothelial cell activation (5–8). In 2024, the FDA released guidance for the industry regarding considerations for the development of CAR-T cell therapies that aims to provide valuable recommendations for CAR-T cell therapy designing, manufacturing, and clinical trial planning (9).

Many clinical trials are ongoing to enhance CAR-T cell therapy’s efficacy, persistence, and safety and decrease relapse rates. These clinical trials either include different combinations of CAR-T cell therapy and other immunotherapy or different/modified CAR constructs (4, 10). In addition, different strategies to extend CAR-T cell persistence and activity against solid tumors are currently under investigation (11–13). Another area of CAR-T cell innovation focuses on allogeneic off-the-shelf cells and *in situ* engineering, addressing the challenges of manufacturing and administration. However, these advancements are beyond the scope of this review. In this review, we provide an overview of the immunogenicity of biologics and its impact on pharmacokinetics (PK), pharmacodynamics (PD), safety, and efficacy. We also discuss the mechanisms of immunogenicity triggered by CAR-T cells and examine clinical evidence related to the immunogenicity of FDA-approved CAR-T products.

### FDA-approved CAR-T cell therapies

Currently, six CAR-T cell products are approved for commercial use by the FDA and are available in the U.S. market for the treatment of different hematological malignancies. Among these six products, four products are for the treatment of B-cell leukemia and lymphoma (Tisagenlecleucel, axicabtagene ciloleucel, lisocabtagene maraleucel, and brexucabtagene autoleucel), two products for the treatment of mantle cell lymphoma (Lisocabtagene maraleucel and brexucabtagene autoleucel), and two products for the treatment of multiple myeloma (MM) (Idecabtagene vicleucel and ciltacabtagene autoleucel) (14). Out of these six CAR-T cell products, tisagenlecleucel, axicabtagene ciloleucel, lisocabtagene maraleucel, and brexucabtagene autoleucel target CD19, which is limited to B cells lineage and is not expressed by pluripotent blood stem cells. Idecabtagene vicleucel and ciltacabtagene autoleucel target BCMA, which present on plasma cells (14).

## Immunogenicity of biologics

Immunogenicity refers to the ability of biologics such as fusion proteins, antibodies, and cellular therapies to induce undesired immune responses against either themselves, related proteins, or to induce immunologically related adverse clinical events (15). The immunogenicity of the biologics can be attributed to their species origin, primary sequence, posttranslational modification, chemical modifications, excipients with adjuvant properties, or impurities during formulation and manufacturing that can be recognized as foreign material by the host immune cells (16). In addition, patient-related factor plays an important role in biologics immunogenicity. For example, patient genetic factors, which predetermine the shape of the immune response, immune status, age, concomitant treatment, and prior exposure to similar proteins can affect the immunogenicity of biologics (3).

Immunogenicity against biologics can impede or terminate the clinical development of such therapeutics (17). For example, an unfavorable safety profile related to immunogenicity may prevent drug approval or require long-term safety and efficacy follow-up which might delay the launch of the product. Although immunogenicity can be assessed *in silico* and *in vitro* to identify potential T cell epitopes during preclinical development, biologic immunogenicity risk still persists during the clinical development phase as immunogenicity of biologics can be attributed to many factors as mentioned earlier. Early identification of the specific factors and underlying immunologic mechanisms of biologics can facilitate the development of strategies to help mitigate their immunogenicity risk. Notably, in certain situations, some biologics demonstrate a favorable benefit-risk balance despite their immunogenicity and get FDA approval (17).

Immunogenicity can be classified as humoral and cellular immunogenicity (18). In clinical practice, humoral immunogenicity (antibody production by B cells) is more frequently measured than cellular immunogenicity (cytotoxic CD4^+^ and CD8^+^ responses) and is assessed by the detection of anti-drug antibodies (ADA) against biologics in the patient serum or plasma (18). In some clinical trials, cellular immunogenicity has been linked to the immune rejection of CAR-T cells, limited CAR-T cell persistence, and treatment failure as described in more detail by Khan et al. (19). In a small study (n=4) treating DLBCL with autologous CD20 CAR-T cells expressing neomycin phosphotransferase, anti-transgene cellular immune responses—though not humoral responses—were observed in two patients, specifically targeting the vector-encoded neomycin phosphotransferase (19, 20). This cellular immune response was associated with the clearance of CAR-T cells in these patients (19, 20). In another study using anti-carbonic anhydrase IX (CAIX) CAR-T cells to treat renal cell carcinoma, both humoral and cellular immune responses were observed in 7 and 9 out of 12 patients, respectively. These immune responses contributed to limited CAR-T cell persistence and eventual clearance (19, 21).

The effects of the produced ADA can vary between no apparent clinical symptoms to life-threatening responses. In most cases, ADA are polyclonal antibodies generally directed against various epitopes of biologics, include multiple isotypes, and circulate at varying concentrations across a diverse array of endogenous proteins (22). Hence, there is a need for sensitive and specific assays to detect ADA in the patient serum or plasma (18). The FDA recommends a 3-tiered approach for ADA characterization (23). Tier 1 is a screening assay (binding antibody assay) that is designed to have high sensitivity for the detection of low levels of ADA in clinical samples (low and high-affinity ADA) (23). Samples with a signal above the cut point (positive) in tier 1, are subjected to tier 2 which is a confirmatory assay (competition assay) to establish the specificity of ADA to biologics (23). Upon confirmation of the presence of ADA in a clinical sample, tier 3 is performed using titration and neutralization assays to characterize the magnitude of the ADA response and its effect on the biologic’s activities (23). This 3-tiered approach minimizes the rate of false positive ADA and characterizes the ADA response associated with biologics. However, the results of these immunogenicity assays are semi-quantitative and the results are dependent on the assay sensitivity and drug tolerance (18). Therefore, the ADA incidence rates or intensity should not be compared between different biologics or for the same biologic when different assays are utilized (18).

### Impact of immunogenicity on pharmacokinetics, pharmacodynamics, efficacy, and safety of biologics

An unwanted immune response against biologics poses a critical risk for the clinical advancement of biologics and the effectiveness of patient therapies. Biologics have complicated structures that result ‘in most cases’ in unique pharmacokinetic and pharmacodynamic (PK/PD) profiles that include nonlinear relationships in the dose-exposure-response curve (24). Immunogenicity associated with biologics makes the *in vivo* predictions of the PK/PD profile more complicated as more variables are included (24). Hence, assessment of biologic’s immunogenicity is required by the regulatory filing agencies for a licensing application to ensure patient safety and effective drug administration (23). The route of administration and the dosage of biologics can influence their immunogenicity. Studies investigating alternative administration routes for infliximab have clearly demonstrated that high-dose regimens are associated with reduced immunogenicity compared to low-dose or interrupted regimens (25).

ADA can affect the PK of biologics (24, 26). For example, ADA bound to therapeutic proteins can affect some of the PK parameters such as the half-life and clearance (24, 26). ADA can extend or shorten the half-time through immune complexes formation that can decrease or increase the biologic’s clearance, respectively (24, 26). Therefore, ADA constitute an additional pathway for therapeutic protein elimination or storage in the body (24, 26). Moreover, ADA bound to biologics can prevent the drug from entering the bloodstream (if not administered *iv*) or site of action (26). The efficacy of the biologics can be affected by ADA as well. ADA can be neutralizing antibodies (nAb) which bind to active/critical sites and inhibit the functional activity of the therapeutic protein leading to the loss of their efficacy (27). Additionally, in some cases, the generation of an immune response against biologics may cross-react with non-redundant essential endogenous proteins leading to loss of their physiological function (16). A well-known example is the generation of nAb against therapeutic erythropoietin that interact and neutralize endogenous erythropoietin as well, leading to pure red cell aplasia (16).

Other major concerns regarding the effect of immunogenicity on the safety of therapeutic protein include injection and acute infusion reactions, anaphylaxis, and hypersensitivity (23). These reactions may develop during (within seconds) or within few hours following infusion (27). For example, pre-existing ADA (IgE) directed against galactose-α-1,3-galactose in the Fab portion of cetuximab lead to fatal infusion reactions (28). Overall, biologic immunogenicity can affect the biologic’s PK, PD, efficacy, and safety. Hence, it can be an obstacle in the way of the clinical development of certain biologics.

## Immunogenicity of CAR-T cell therapies

Even though CAR-T cell therapies are derived from the patient’s own cells, they can still induce unwanted immune responses. These responses are not caused by post-translational modifications or protein aggregates, which are factors that could contribute to the immunogenicity of other biologics. Immune responses against CAR-T cell therapies can be elicited because of the non-self-component of CAR-T cells (mouse or humanized scFv), linker proteins, hinge and transmembrane domain, co-stimulatory domains, residual proteins from CAR transfer vector, impurities that have adjuvant properties, or pre-exposing to mouse monoclonal antibodies through different medications (3, 29). Immunogenicity against CAR-T cells can be cellular and/or humoral, although innate immune response could also be stimulated to facilitate the break of immune tolerance (3, 29). The produced ADA can bind to CAR-T cells and alter their PK/PD profile, safety, and efficacy.

The antibody response is further characterized by different ADA categories including pre-existing ADA (antibody present before treatment), treatment-emergent ADA (antibody developed following drug administration in subjects without pre-existing ADA, or pre-existing ADA were boosted to a higher level with ADA titer greater than the baseline titer after treatment), persistent ADA (based on duration of ADA response), etc. While the humoral response as measured by ADA formation is more studied than the cellular response, both responses could affect the overall PK, PD, clinical safety, and efficacy of CAR-T cell therapies (27). In addition, cellular immunogenicity can induce long-term memory cells that can affect the efficacy during retreatment or treatment with other CAR-T therapies with shared components/sequences (29). To get more details about immunogenicity assays, immunogenicity risk factors, mitigation of immunogenicity, and recommendations for assay deployment, the readers are encouraged to read Gokemeijer et al. and Mody et al. (18, 29).

### Cellular arm of immunogenicity against CAR-T cell therapies

**Figure 1:**
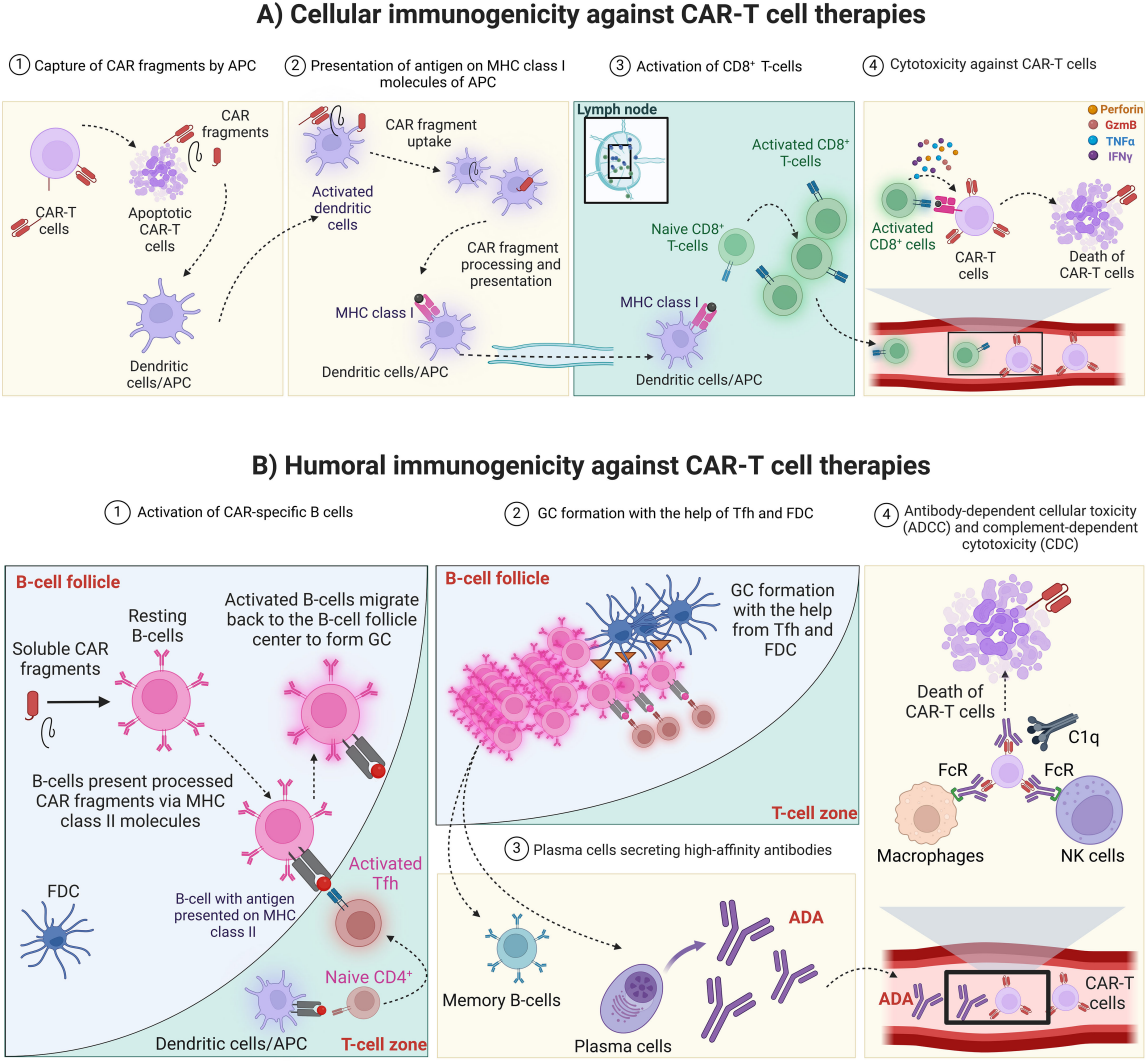
CAR-T cell therapy immunogenicity. **(A)** Cellular immunogenicity against CAR-T cell therapies. 1) Apoptotic or necrotic CAR-T cells release CAR fragments that can be captured by APC such as dendritic cells and macrophages. 2) APC capture, process and present CAR fragments on MHC class I and II molecules (MHC class II is not shown for simplicity) then travel to secondary lymphoid organ such as the lymph nodes. Alternatively, soluble antigen can arrive to the lymph nodes and be up taken, processed and presented on MHC molecules of resident dendritic cells. 3) In the lymph node, APC can activate cytotoxic T cells that recognize the antigen presented by MHC molecules. 4) In the circulation, cytotoxic T cells identify and interact with CAR-T cells that present the CAR antigen on their MHC molecules. CAR, Chimeric antigen receptor; CAR-T cell, Chimeric antigen receptor-T cell; MHC, Major histocompatibility complex; APC, Antigen-presenting cells; GzmB, Granzyme B. Created with BioRender.com. **(B)** Humoral immunogenicity against CAR-T cell therapies. 1) In the lymph node, naïve follicular B cells encounter antigenic CAR peptides and become activated. The activated B cells present processed peptide on MHC class II molecules to Tfh cells which have been primed by APC presenting the specific Th2 epitope generated from CAR peptide. The interaction between Tfh and follicular B-cells results in the full activation of each other. 2) Later on, fully activated B-cells migrate to the center of B-cell follicle to form GC, where B-cells proliferate rapidly with simultaneous somatic hypermutation and clonal selection, with the help from Tfh and FDC, to increase their affinity for antigen 3) GC reaction produces both plasma cells and memory B-cells that produce high-affinity antibodies (ADA). 4) ADA bind to CAR-T cells and mediate their cytotoxicity through antibody-dependent cellular toxicity (ADCC) or complement-dependent cytotoxicity (CDC). CAR, Chimeric antigen receptor; MHC, Major histocompatibility complex; APC, Antigen-presenting cells; FDC, Follicular dendritic cells; Tfh, Follicular T helper cells; GC, Germinal center; ADA, Anti-drug antibodies; FcR, Fc receptors; C1q, Component 1q; NK cells, Natural killer cells. Created with BioRender.com.

Antigen-presenting cells (APC) such as macrophages and dendritic cells are professional immune cells that are equipped with receptors that allow them to interact, destroy and phagocytose foreign molecules and present them to lymphocytes (**Figure 1A**) (3, 30). The CAR peptides from apoptotic or necrotic CAR-T cells can be up-taken by APC and presented on the MHC molecules on their surfaces (**Figure 1A**) (3). APC can prime both cytotoxic CD8^+^ and CD4^+^ T cells through antigens presented on MHC class I and II, respectively (3). The activated cytotoxic T cells can then recognize the T cell epitope presented by MHC molecules of the CAR-T cells and release their soluble factors such as cytokines, interferons, perforin, and granzymes resulting in CAR-T cell death (**Figure 1A**) (3). The cellular immunogenicity is less studied than humoral immunogenicity due to the technical challenges in developing a robust and sensitive cellular immune assay and the lack of a full understanding of the impact of cellular immunogenicity in clinical settings. A proposed approach to detect cellular immunogenicity involves the collection and freezing of Peripheral Blood Mononuclear Cells (PBMCs) at key time points during treatment. This strategy helps address safety and efficacy concerns that may arise in specific patients, especially when clinical endpoints fail to provide clear explanations. Tisagenlecleucel is the only CAR-T cell therapy that was tested for clinical cellular immunogenicity according to the package insert (31).

### Humoral arm of immunogenicity against CAR-T cell therapies

The fully activated humoral response relies on the interaction between follicular B cells and follicular T-helper cells (Tfh). In the lymph nodes, naïve follicular B cells encounter antigenic CAR peptides and become activated (32–35). The activated B cells present processed peptides on MHC class II molecules to Tfh cells which have been primed by APC presenting the specific T helper cell (Th2) epitope generated from CAR peptide (**Figure 1B**) (32–35). The interaction between Tfh and follicular B cells results in the full activation of each other (32–35). Some of the fully activated B cells then differentiate to plasmablasts producing low-affinity antibodies, whereas a subset of these activated B cells migrates to the center of the B cells follicle and form a germinal center (GC) (32–35). In GC, B cells proliferate rapidly with simultaneous somatic hypermutation and clonal selection, with the help of Tfh and follicular DC (FDC), to increase their affinity for antigen (32–35). GC reaction produces both plasma cells and memory B cells that produce long-lasting and high-affinity antibodies (ADA) (**Figure 1B**) (32–35). The produced ADA can bind to CAR-T cells, neutralize their function, and mark them for clearance by antibody-dependent cytotoxicity (through the Fc receptor of the innate immune cells), or through complement-dependent cytotoxicity (**Figure 1B**) (3). In addition, the presence of anti-CAR IgE could result in mast cell-mediated cytotoxicity and result in severe systemic anaphylaxis (3).

## Clinical evidence of CAR-T immunogenicity

In clinical settings, the humoral immunogenicity of CAR-T cell therapies is revealed based on ADA detection in the patient serum or plasma. However, the cellular immunogenicity is much less studied during clinical trials. From the currently approved CAR-T cell therapies, one drug (tisagenlecleucel only) was evaluated for cellular immunogenicity according to the package insert (31). In five out of six FDA-approved CAR-T cell therapies, ADA were detected at least either before or after CAR-T cell infusion (18). However, there is no clear evidence showing the presence of ADA has any impact on PK/PD profile or induces ADA-related toxicity in patients (3, 29). This might be due to the lymphodepletion chemotherapy administered before CAR-T cell infusion, which is thought to enhance CAR-T cell persistence and efficacy by creating a more favorable environment for the infused cells. Lymphodepletion can also reduce the host immune response, potentially minimizing the immunogenicity of the CAR-T cells. Additionally, CAR-T cell therapies targeting B cells may further minimize immunogenicity by reducing the number of immune cells that could otherwise react against the infused therapy (18). Another potential explanation might be the discrepancy in CAR-T cell expansion and the time it takes to form cellular and humoral immune responses against the infused CAR-T cells. In most cases, CAR-T expansion occurs in about two weeks and the onset of the immune response varies between 3-6 months, which does not impact the expansion phase of CAR-T cells (29). However, the humoral immunogenicity of some CAR-T cell therapies in clinical development affected the PK/PD profile of these therapies (18, 36–38).

Since clinical trials are conducted under different conditions, patient populations, and assay settings, the ADA rates observed in clinical trials of a CAR-T cell therapies cannot be directly compared to the ADA rates of other CAR-T cell therapies (18). In this section, we describe a brief summary of all of the FDA-approved CAR-T cell therapies and their immunogenicity. **Table 1** contains a concise summary of each therapy.

**Table 1:** Summary of the currently FDA-approved CAR-T cell therapies.

Drug (Commercial name)	CAR construct	Clinical trials	Indications	Dosing regimens	Pre-existing ADA	Treatment-emergent ADA	Cellular immunogenicity	Impact of immunogenicity on PK/efficacy/safety
Tisagenlecleucel (KYMRIAH)	Anti-CD19, CD8α hinge and transmembrane regions, 4–1BB (CD137), and CD3ζ chain	ELIANA; NCT02435849	r/r B-ALL	For patients 50 kg or less, 0.2 to 5.0 × 10^6 CAR-positive viable T cells/kg. For patients above 50 kg, 0.1 to 2.5 × 10^8 total CAR-positive viable T cells	91%	42%	<1% CD4^+^ and CD8^+^ activation and IFNγ production	No effect on initial expansion, persistence, safety or effectiveness
		JULIET; NCT02445248	r/r DLBCL		94%	9%		
		ELARA; NCT03568461	r/r FL	Dose range was 0.1 to 6.0 × 10^8 CAR-positive viable T cells	66%	33%		
Axicabtagene ciloleucel (YESCARTA)	Anti-CD19, CD28α hinge and transmembrane regions, CD28, and CD3ζ chain	ZUMA-1; NCT02348216	r/r LBCL	Dose: 2 × 10^6 CAR-positive viable T cells/kg (maximum permitted dose: 2 × 10^8 CAR-positive viable T cells)	0%	0%	NA	NA
		ZUMA-5; NCT03105336	r/r FL		0%	0%		
		ZUMA-7; NCT03391466	r/r LBCL		0%	0%		
Brexucabtagene autoleucel (TECARTUS)	Anti-CD19, CD28α hinge and transmembrane regions, CD28, and CD3ζ chain	ZUMA-2; NCT02601313	r/r MCL	Dose range of 1 to 2 × 10^6 CAR-positive viable T cells/kg	0%	0%	NA	No effect on initial expansion, persistence, safety or effectiveness
		ZUMA-3; NCT02614066	r/r B-ALL	Dose: 1 × 10^6 CAR-positive viable T cells/kg (maximum 1 × 10^8 CAR-positive viable T cells)	0%	2%		
Lisocabtagene maraleucel (BREYANZI)	Anti-CD19, IgG4 hinge region, CD28 transmembrane domain, 4–1BB (CD137), and CD3ζ chain	TRANSFORM; NCT03575351	r/r LBCL	Dose: 1 × 10^8 CAR-positive viable T cells	1%	1%	EXPLORATORY DATA Very low or undetectable levels of IFNγ release measured by ELISA	No conclusion on the impact of immunogenicity on cellular kinetics, clinical response, or safety
		PILOT; NCT03483103	r/r LBCL		0%	2%		
		TRANSCEND-CLL; NCT03331198	r/r CLL or SLL		2%	7%		
		TRANSCEND-FL; NCT04245839	r/r FL		2%	18%		
		TRANSCEND-MCL Cohort; NCT02631044	r/r MCL		13%	18%		
		TRANSCEND; NCT02631044	r/r LBCL	Dose range of 50 to 110 × 10^6 CAR-positive viable T cells	11%	11%		
Idecabtagene vicleucel (ABECMA)	Anti-BCMA, CD8α hinge and transmembrane domain, 4–1BB (CD137), and CD3ζ chain	KarMMa; NCT03361748	r/r MM	Dose range of 150 to 518 × 10^6 CAR-positive viable T cells	2.60%	53%	EXPLORATORY DATA Undetectable levels of IFNγ release from PBMC by ELISPOT	No effect on initial expansion, persistence, safety or effectiveness
		KarMMa-3; NCT03651128		Dose range of 175 to 529 × 10^6 CAR-positive viable T cells				
Ciltacabtagene autoleucel (CARVYKTI)	Dual linked VHH anti-BCMA, CD8α hinge and transmembrane domain, 4–1BB (CD137), and CD3ζ chain	CARTITUDE-1; NCT03548207	r/r MM	Dose range of 0.51 to 0.95 × 10^6 CAR-positive viable T cells/kg	NA	19.60%	NA	No evidence to suggest an impact on drug exposure, efficacy or safety
		CARTITUDE-4; NCT04181827	relapsed and lenalidomide-refractory MM	Dose range: 0.39 to 1.07 × 10^6 CAR-positive viable T cells/kg	21% either pre or post CARVYKTI infusion			

r,r, relapse or refractory; B-ALL, B-cell acute lymphoblastic leukemia; DLBCL, diffuse large B-cell lymphoma; FL, Follicular lymphoma; LBCL, large B-cell lymphoma; MCL, mantle cell lymphoma; CLL, chronic lymphocytic lymphoma; SLL, small lymphocytic lymphoma; MM, multiple myeloma. NA, Not Available.

### Tisagenlecleucel (KYMRIAH™)

Tisagenlecleucel is anti-CD19 CAR-T cell therapy commercially known as KYMRIAH™ and sold by Novartis (31). Tisagenlecleucel is used for the treatment of relapsed or refractory (r/r) acute lymphoblastic leukemia (r/r ALL), r/r diffuse large B-cell lymphoma (r/r DLBCL), and follicular lymphoma (FL) (31). Treatment with tisagenlecleucel must be preceded by lymphodepletion therapy with fludarabine and cyclophosphamide unless the patient has cytopenia (WBC ≤ 1 x 10^9^/L) within 1 week prior to tisagenlecleucel infusion (31). Tisagenlecleucel is generated from CD4^+^/CD8^+^ T cell enrichment of each patient peripheral blood mononuclear cells (PBMCs), which are then transduced with a replication-incompetent self-inactivating lentiviral vector (LV) that has an anti-CD19 CAR transgene (39). Tisagenlecleucel consists of a murine scFv of FMC63 monoclonal antibody that binds to CD19 on tumor cells and is fused to the CD8α hinge and transmembrane regions followed by the costimulatory molecule 4–1BB (CD137) and the T cell activation domain CD3ζ chain (31, 40). Since tisagenlecleucel contains a murine scFv, it has a higher risk of eliciting an immune response in patients. However, no preclinical studies were done to test the immunogenicity of tisagenlecleucel. The preclinical efficacy studies of tisagenlecleucel against leukemia were done at the University of Pennsylvania using an immunodeficient xenograft mouse model (NSG mice) engrafted with tumor cells from patients with ALL and not from patients with DLBCL or FL (41, 42).

During the three main clinical studies of tisagenlecleucel (ELIANA, JULIET, and ELARA), preexposure and post-exposure serum samples (at days 14 and 28; at months 3, 6, 12, and 24 (collected at month 36 in ENSIGN only); and upon relapse)) were collected from participating patients to assess the humoral immunogenicity before and after tisagenlecleucel (31, 43). Cellular-based assays were used during the screening and confirmatory assays for ADA detection (18, 31). More details regarding ADA detection assays against tisagenlecleucel can be found in (44). Most patients, 91% in ELIANA (r/r B-cell ALL), 94% in JULIET (r/r DLBCL), and 66% in ELARA (r/r FL), tested positive for pre-dose anti-mCAR19 antibodies before tisagenlecleucel infusion (31). Posttreatment ADA were higher than the patient-specific baseline in 42%, 9%, and 33% of the patients in ELIANA, JULIET, and ELARA, respectively (31, 43). However, the preexisting and treatment-emergent ADA were not associated with any impact on clinical responses (day-28 response, duration of response, and event-free survival), cellular kinetics (maximum concentration and persistence), safety (CRS, neurologic events, or susceptibility to infection) or the initial expansion and persistence of tisagenlecleucel (31, 43). The absence of impact on efficacy provides a rationale for not performing neutralizing antibody assessments for tisagenlecleucel. The cellular immunogenicity was determined using IFNγ release from T cells in response to 2 different pools of mCAR19 peptides using intracellular staining of IFNγ followed by flow cytometry detection (43). The percentage of T cells activated pre- and post-tisagenlecleucel infusion (up to 12 months) was calculated and the cellular immunogenicity was consistently low (∼1%) over time for individual patients (31, 45). Hence, the cellular immunogenicity (CD4^+^ and CD8^+^ responses) did not affect tisagenlecleucel transgene expansion, persistence, or patient outcomes (43).

### Axicabtagene ciloleucel (YESCARTA™)

Axicabtagene ciloleucel is an anti-CD19 CAR-T cell therapy commercially known as YESCARTA™ and sold by Kite (a Gilead company) (46). Axicabtagene is used for the treatment of adult patients with r/r large B-cell lymphoma (LBCL), and r/r FL (46). Treatment with axicabtagene must be preceded by lymphodepletion therapy with fludarabine and cyclophosphamide (46). Axicabtagene is generated from CD3^+^ enriched autologous T cells that are transduced with a replication-incompetent γ retroviral vector containing an anti-CD19 CAR (39, 40). Axicabtagene contains a murine extracellular scFv of the FMC63 monoclonal antibody that binds to CD19 on tumor cells followed by a human CD28α hinge and transmembrane domain fused to the costimulatory molecule CD28 and the T cell activation domain CD3ζ chain (40). No preclinical studies were done to test the immunogenicity of axicabtagene.

During the main clinical studies of axicabtagene (ZUMA-1,5, and 7), preexposure and post-exposure serum samples were collected from participating patients to assess the humoral immunogenicity before and after axicabtagene infusion (46). Humoral immunogenicity was assessed by an ELISA-based assay against the murine monoclonal antibody FMC63, the parent antibody from which the scFv utilized in axicabtagene was developed (46, 47). Initial ELISA screening in patients in ZUMA-1 and ZUMA-7 studies has shown that 4% (11 patients) were ADA positive at baseline before lymphodepletion chemotherapy (3 patients from ZUMA-1 and 8 patients from ZUMA-7) and 1% (one patient) in ZUMA-7 who had negative test results at baseline developed ADA post axicabtagene infusion (46, 47). In ZUMA-5, 13% (19 patients) of the patients were ADA positive at baseline and 2% (3 patients) who had negative test results at baseline developed ADA post axicabtagene infusion (46). However, all patients were ADA negative at all time points tested when assessed with a confirmatory cell-based flow cytometry assay using a properly folded and expressed extracellular portion of the CAR construct (scFv, hinge, and linker) (46, 47). No data were reported regarding the cellular immunogenicity of axicabtagene.

### Brexucabtagene autoleucel (TECARTUS™)

Brexucabtagene autoleucel is anti-CD19 CAR-T cell therapy commercially known as TECARTUS™ and is indicated for the treatment of adult patients either with r/r mantle cell lymphoma (MCL) or ALL (48). Treatment with brexucabtagene must be preceded by lymphodepletion therapy with fludarabine and cyclophosphamide (49). Brexucabtagene is sold by Kite and generated and contains the same component as axicabtagene (14). Brexucabtagene contains a murine extracellular scFv of the FMC63 monoclonal antibody that binds to CD19 on tumor cells followed by a human CD28α hinge and transmembrane domain fused to the costimulatory molecule CD28 and the T cell activation domain CD3ζ chain (40). The main difference between brexucabtagene and axicabtagene is an extra step in the production of brexucabtagene that aims to remove malignant cells from the leukapheresis products (14). No preclinical studies were done to test the immunogenicity of brexucabtagene. The preclinical efficacy studies of brexucabtagene were done in a syngeneic mouse lymphoma model (50).

During the main clinical studies of brexucabtagene (ZUMA-2 and 3), preexposure and post-exposure serum samples were collected from participating patients to assess the humoral immunogenicity before and after brexucabtagene (48). Humoral immunogenicity was assessed by an ELISA-based assay against the murine monoclonal antibody FMC63, the parent antibody from which the scFv utilized in brexucabtagene was developed (48). During the screening assay for patients in ZUMA-2,~21% of the patients (17 of 82 patients) tested positive for ADA at any time point (48). However, the screening results were false positive results that were revealed to be negative upon doing a confirmatory cell-based assay using a properly folded scFv expressed on the surface of an anti-CD19 CAR-T cell (48). In ZUMA-3, the ADA screening assay revealed that 16% (16 of 100 patients) tested positive for ADA at any timepoint (48). The confirmatory assay was done on patients with evaluable samples (48). Among them, only two subjects were confirmed to be ADA positive after brexucabtagene infusion (48). One of the two patients had a confirmed positive ADA at Month 6 (48). The second patient had a confirmed ADA result at retreatment Day 28 and Month 3 (48). ADA have no effect on initial expansion, persistence, safety or effectiveness of brexucabtagene (48).

### Lisocabtagene maraleucel (BREYANZI™)

Lisocabtagene maraleucel is an anti-CD19 CAR-T cell therapy commercially known as BREYANZI™ for the treatment of adult patients with LBCL, r/r lymphocytic leukemia (r/r CLL) or small lymphocytic lymphoma (r/r SLL), r/r FL, and r/r MCL (51). Treatment with lisocabtagene must be preceded by lymphodepletion therapy with fludarabine and cyclophosphamide (51). Lisocabtagene is generated from CD4^+^/CD8^+^ T cell enrichment of PBMCs and each population (CD4^+^ and CD8^+^) is transduced with a replication-incompetent self-inactivating LV containing an anti-CD19 CAR (40). This allow the delivery of defined CD4^+^:CD8^+^ T cell composition (1:1), which enhance total cell growth as compared to CD8^+^ subset alone (14, 39). Lisocabtagene is comprised of a murine extracellular scFv of the FMC63 monoclonal antibody that binds to CD19 on tumor cells followed by a human IgG4 hinge region, CD28 transmembrane domain fused to the costimulatory molecule 4–1BB (CD137), and the T cell activation domain CD3ζ chain (40). In addition, lisocabtagene has a nonfunctional truncated epidermal growth factor receptor (EGFRt) that is co-expressed on the modified T cell surface with the CD19-specific CAR (51). The addition of the EGFRt can be used for rapid elimination of CAR-T cells in patients who express lisocabtagene infusion toxicity through the administration of cetuximab (Erbitux^®^) (39). EGFRt expression might also help in the selection/tracking of transduced cells by flow cytometry using anti-EGFR monoclonal antibody such as cetuximab or by EGFRt immunomagnetic positive selection (52, 53). However, this addition might be associated with an increased risk of lisocabtagene immunogenicity. No preclinical studies were done to test the immunogenicity of lisocabtagene. The preclinical efficacy studies of lisocabtagene were done using immune-deficient NSG mice engrafted human CD19^+^ Raji Burkitt’s lymphoma cells to demonstrate the proof of principle of the therapy (42, 54).

During the main clinical studies of lisocabtagene (TRANSCEND, TRANSCEND-CLL, TRANSCEND-FL, TRANSCEND-MCL, TRANSFORM, and PILOT) preexposure and post-exposure samples (serum and plasma) were collected from participated patients to assess the humoral immunogenicity before and after lisocabtagene infusion (51, 54, 55). The formation of ADA against the extracellular domain of lisocabtagene was evaluated in plasma and serum by an ECL-based immunoassay (51). Pre-existing ADA were detected in 11% (28/261 patients) in TRANSCEND, 2% (2/86 patients) in TRANSCEND-CLL, 2% (2/102 patients) in TRANSCEND-FL, 13% (11/87 patients) in TRANSCEND-MCL, 1% (1/89 patients) in TRANSFORM, and 0% (0/51 patients) in PILOT (51). Treatment induced or treatment-boosted ADA were detected in in 11% (27/257 patients) in TRANSCEND, 7% (6/84 patients) in TRANSCEND-CLL, 18% (18/100 patients) in TRANSCEND-FL, 18% (15/85 patients) in TRANSCEND-MCL, 1% (1/89 patients) in TRANSFORM, and 2% (1/49 patients) in PILOT (51). In the TRANSFORM study, only one patient with preexisting ADA achieved a best overall response (BOR) of complete response (CR) and did not experience any CRS or neurotoxicity (54, 55). The patient’s Cmax and AUC (0-28 days) were lower than the median values of the overall lisocabtagene arm patients (54, 55). In another patient who had a treatment-induced ADA in the same study, BOR of a CR was achieved without CRS or neurotoxicity (54, 55). The patient Cmax and AUC (0-28 days) were higher than the median values of overall population (55). However, no conclusion was made regarding the impact of preexisting and treatment-induced ADA on clinical response, cellular kinetics, safety, or the initial expansion and persistence of lisocabtagene (51, 55). The cellular immunogenicity of lisocabtagene was measured using ELISA to detect IFNγ release from T cells in response to the extracellular domain (ECD) of lisocabtagene (54). The patient samples exhibit very low or undetectable levels of IFNγ release from a single T cell (54). However, the cellular immunogenicity analysis was considered exploratory only and not intended to support any interpretation of the clinical observations of lisocabtagene (54). The absence of impact on efficacy provides a rationale for not performing neutralizing antibody assessments for lisocabtagene.

### Idecabtagene vicleucel (ABECMA™)

Idecabtagene vicleucel is an anti- B-cell maturation antigen (BCMA) genetically modified autologous T cell immunotherapy for the treatment of adult patients with r/r MM (56). Treatment with idecabtagene must be preceded by lymphodepletion therapy with fludarabine and cyclophosphamide (56). Idecabtagene is generated from CD3^+^ enriched autologous T cells that are transduced with a replication-incompetent self-inactivating LV that has anti-BCMA CAR transgene (40). Idecabtagene is comprised of a murine extracellular scFv of the C11D5.3 monoclonal antibody that binds to BCMA on tumor cells followed by a human CD8α hinge and transmembrane domain fused to the costimulatory molecule 4–1BB (CD137) and the T cell activation domain CD3ζ chain (14, 57). No preclinical studies were done to test the immunogenicity of idecabtagene. The preclinical efficacy studies of idecabtagene were done using immunodeficient mice NSG with human tumor cell line xenografts (58, 59).

During the main clinical studies of idecabtagene (CRB-401, KarMMa [MM-001], and KarMMa-3[MM-003]) preexposure and post-exposure serum samples were collected from participated patients to assess the humoral immunogenicity before and after idecabtagene infusion using a validated immunoassay (ECL-MSD) to detect ADA against the extracellular CAR domain of idecabtagene (56, 59). In the Phase 1 study (Study No. CRB-401), 5.4% of patients had pre-existing ADA before idecabtagene infusion (59). Approximately 3.8% (2 of 52 patients), 40.9% (18 of 44 patients), 61.8% (21 of 34 patients), 65.2% (15/23 patients), and 80% (12/15 patients) were ADA positive by Month 1, Month 3, Month 6, Month 9, and Month 12 post-infusion, respectively (59). In the pivotal phase 2 study (Study No. BB2121-MM-001), there was <5% of the patients with pre-existing ADA before idecabtagene infusion (60). ADA did not develop in the first month post-infusion of idecabtagene (0/123) (59). However, at sampling visits; Month 3, Month 6, Month 9, and Month 12 after infusion, approximately 20.6% (21 of 102 patients), 43.8% (35 of 80 patients), 57.8% (37 of 64 patients), and 62.1% (18 of 29 patients) patients were ADA positive, respectively (59). During the phase 3 trial (MM-003), 2.3% patients had preexisting ADA (5 of 219 patients) (61). At sampling visits; Day 25, Month 2, Month 4, Month 6, Month 10, Month 19, and Month 31; ADA % were 1% (2 of 207 patients), 0.9% (2 of 217 patients), 22.9% (41 of 179 patients), 50.6% (84 of 166 patients), 71.4% (85 of 119 patients), 92.9% (26 of 28 patients), and 100% (3 of 3 patients), respectively (61). Exposure variables (AUC0−28 days and Cmax) in ADA positive patients were comparable to the overall study population (59–61). Since the majority of subjects did not develop ADA in the first-month post-infusion and the expansion of the CAR-T cells occurred mainly within one month (with the peak expansion occurring at a median of 11 days), the ADA response is not likely to have an impact on the cell expansion and PK of idecabtagene (56, 59–62). This is confirmed by a univariate analysis by which ADA status was not found to influence the cellular kinetics, whereas body weight, baseline soluble BCMA level, memory T cell status (CD3+CAR+CCR7+CD27), vector copy number, and a few other attributes were associated with the PK parameters (61). No discernible difference in transgene levels was seen between ADA positive and ADA negative patients through Month 1 post-infusion; however, by Month 5 Day 1, “the median transgene level in ADA positive patients was considerably lower than that of ADA negative subjects” (61). In addition, the development of ADA did not increase the frequency or severity of CRS or neurotoxicity or safety of idecabtagene (59, 61). Therefore, the presence of ADA did not appear to have a clinically significant impact on PK, safety or efficacy (56, 59–62).

The final reported ADA % against idecabtagene was combined from two clinical trials (KarMMa [MM-001] and KarMMa-3[MM-003]) (56). Around 2.6% of patients tested positive for pre-infusion ADA and treatment-induced ADA were detected in 53% of the patients (56). The cellular immunogenicity of idecabtagene was evaluated using an IFNγ ELISPOT assay from PBMCs derived from subjects and stimulated *ex vivo* using peptides spanning the ECD of the CAR construct during KarMMa [MM-001] study and IFNγ release was not detected (59, 62). This data was not mentioned in the package insert as it was supportive data and not intended to be used to support any clinical claims of the cellular immunogenicity of idecabtagene (59, 62). The absence of impact on efficacy provides a rationale for not performing neutralizing antibody assessments for idecabtagene.

### Ciltacabtagene autoleucel (CARVYKTI™)

Ciltacabtagene autoleucel is anti-BCMA modified autologous T cell immunotherapy for the treatment of adult patients with r/r MM (63). Treatment with ciltacabtagene must be preceded by lymphodepletion therapy with fludarabine and cyclophosphamide (63). Ciltacabtagene is generated from CD3^+^ T cells that have been transduced using a replication-incompetent self-inactivating LV that has anti-BCMA CAR transgene (40). Ciltacabtagene is comprised of dual-linked camelid heavy-chain-only variable (VHH) antigen-binding domains against BCMA followed by a human CD8α hinge and transmembrane domain fused to the costimulatory molecule 4–1BB (CD137) and the T cell activation domain CD3ζ chain (14). The use of the dual-linked camelid increases the avidity and might decrease the immunogenicity as the light chain and the synthetic linker peptides are missing (14, 64, 65).

No preclinical studies were done to test the immunogenicity of ciltacabtagene. The preclinical efficacy studies of ciltacabtagene were done using a MM xenograft model of NCG mice as a proof of concept (66). Moreover, a non-GLP safety study on cynomolgus monkeys using autologous CAR-T cells was performed (66). However, ciltacabtagene didn’t bind to cynomolgus BCMA, which makes the cynomolgus monkey model irrelevant to evaluate potential safety risks of ciltacabtagene in humans (66).

During the two main clinical studies of ciltacabtagene (CARTITUDE-1 and CARTITUDE-4), preexposure and post-exposure serum samples were collected from participating patients to assess the humoral immunogenicity before and after ciltacabtagene using a validated immunoassay to detect ADA against the extracellular BCMA-binding domain of ciltacabtagene (63). In the CARTITUDE-1 study, 19.6% (19 of 97 patients) were positive for treatment emerged ADA (67). In CARTITUDE-4 study, 21% of the patients (39 of 186 patients) were ADA positive (63, 68). The occurrence rates of CRS or CAR-T cell-related neurotoxicity were similar between ADA positive and ADA negative subjects (67, 68). ADA started to be detectable around Day 100 post ciltacabtagene infusion (67). No conclusion was made regarding the effect of ADA on the initial expansion and persistence, efficacy, or safety of ciltacabtagene (63). The absence of impact on efficacy provides a rationale for not performing neutralizing antibody assessments for ciltacabtagene.

## Preclinical animal models to study immunogenicity of CAR-T cells

Before moving a drug candidate into humans, preclinical testing is required by the FDA in most cases. The FDA requires extensive pharmacology, toxicology, and safety testing *in vitro* and/or in animal models before starting human clinical trials. However, CAR-T cell therapy is a special case as it consists of living drugs that can proliferate, expand, migrate, and persist in patients for a long time. Therefore, designing a preclinical model that provides comprehensive pharmacology, toxicology, and safety data is challenging.

Animal models have been useful to assess the efficacy of CAR-T cell therapy in tumor eradication. Most preclinical efficacy studies on CAR-T cell therapy have been conducted in xenograft mice, which failed to predict CAR-T cell-associated immunogenicity and toxicity (42). Newer models such as syngeneic, transgenic, humanized mouse models, and primate models have been developed to assess toxicity and various concerns associated with CAR-T therapy, for example, CRS and neurotoxicity, off-target toxicity, on-target, off-tumor toxicity, Graft-versus-Host Disease (GVHD), and rejection (69, 70). In addition, *in vitro* models such as mixed lymphocyte reactions by coculturing CAR-T cells with PBMCs of different donors could be used to predict the immune response induced by CAR-T cells (3, 71). However, none of these models can perfectly mirror the complexities of the human immune system or accurately anticipate CAR-T cell therapy adverse reactions, but they can provide some insights about potential immunogenicity in clinical trials (70).

The humanized mouse model is a promising tool to assess the immunogenicity of CAR-T cell therapies (69, 70). This model uses immunocompromised mice to allow human immune cells, tumor, and CAR-T cell engrafting (69, 70, 72). It allows the study of human immune and CAR-T cell interaction, predicts CRS, and neurotoxicity observed in patients after CAR-T cell infusions, on-target off-tumor reactions, and potential rejection of infused CAR-T cells (69, 70, 72). In a recent study, humanized mice reconstituted with human CD34^+^ cord blood-derived hematopoietic stem cells (HSC), were used to assess the persistence and efficacy of allogeneic hypoimmunogenic anti-CD19 CAR-T cell therapy (HIP CAR-T) (73). HIP CAR-T are T cells isolated from healthy donors that are modified to knock out TRAC, B2M, and CIITA to eliminate the expression of the endogenous T cell receptor (TCR) and human leukocyte antigens (HLA) class I and II (88.4% and 83.7% negative for HLA class I or II, respectively) (73). Additionally, these cells are modified to express CD47 to protect them against innate immune cell killing (73). Compared to CD19 allogeneic CAR-T, HLA^-^ HIP CAR-T cells showed persistent efficacy in fully immunocompetent humanized mice (73). Cellular immunogenicity was evaluated using IFNγ ELISPOT and cytotoxicity assays (73). It was found that HLA-expressing cells in the HIP CAR-T bulk cells were still able to immunize the host and induce an HLA-directed response in these models, however, sorted HLA^−^ cells from the HIP CAR-T cell bulk did not induce any immune response (73). The humanized mouse model used in this study provided insights about the immunogenicity of allogeneic CAR-T and despite having some limitations such as the lack of human stroma, inadequate establishment of the human immune system in mice, high costs, laborious and long engraftment times, it could be a valuable tool for preclinical immunogenicity assessment for CAR-T therapy in the future (69, 70, 72).

## Conclusions

Similar to other biologics CAR-T cell therapies have the concern of immunogenicity. This is attributed to the non-self-component of CAR-T cells, linker proteins, residual proteins from the CAR transfer vector, or impurities that have adjuvant properties. Immunogenicity against CAR-T cell therapy can be humoral or cellular. Both responses could impact the PK, PD, efficacy, and safety of CAR-T cell therapies. With the genetic engineering of more complex CAR and development of both autologous and allogeneic CAR-T therapies, the challenge of immunogenicity needs careful evaluation, and the strategy to mitigate and monitor the immunogenicity of CAR-T needs to be incorporated into the development of these unique drug products. However, none of the currently FDA-approved CAR-T cell therapies are associated with any type of immunogenicity that could affect the PK/PD profile or patient safety. This might be attributed to the lymphodepletion step that is done before CAR-T cell infusion and the fact that treatment-emergent ADA generally appeared at a later stage than the expansion phase of CAR-T cells *in vivo*, which could explain the lack of effect of immunogenicity on the cellular kinetics and clinical response of CAR-T cell therapies. Across the six approved CAR-T products, the frequencies of pre-existing ADA vary from 0% to 94%, however, these numbers should be interpreted with caution due to the different bioanalysis assays used in these clinical studies.

The cellular immunogenicity of CAR-T cell therapies was less studied than ADA response due to the technical challenges in developing a robust and sensitive cellular immune assay, and the lack of understanding of the impact of cellular immunogenicity in clinical setting. One of the proposed approaches is to collect and freeze the PBMCs at only relevant and potentially informative, and only have them tested when serious efficacy or safety concerns arise for a specific patient and cannot be explained by ADA response or other clinical endpoints (15).

The development of new CAR-T products such as allogeneic CAR-T and armored CAR-T cells present more challenges for the clinical assessment of immunogenicity. The foreign antigens in CAR construct, the armored cytokines, MHC, and other molecules with polymorphism in allogenic cells all present risks of immunogenicity and may warrant different bioanalytical assays to assess the humoral and/or cellular immune responses against these components. Therefore, a risk-based monitoring and bioanalytic strategy needs to be in place during clinical development with consideration of different risk factors, technical and logistic issues, and clinical impact. However, several tools are currently available to evaluate the immunogenicity risks of CAR-T therapies before reaching the clinical phase, such as *in silico* prediction of T cell epitopes, *in vitro* T cell proliferation assay, and preclinical animal models such as humanized mouse models, which will together reduce the potential immunogenicity and help develop a cellular product with improved efficacy and safety profile.
